# Pathophysiological Significance of Dermatan Sulfate Proteoglycans Revealed by Human Genetic Disorders

**DOI:** 10.3390/ph10020034

**Published:** 2017-03-27

**Authors:** Shuji Mizumoto, Tomoki Kosho, Shuhei Yamada, Kazuyuki Sugahara

**Affiliations:** 1 Department of Pathobiochemistry, Faculty of Pharmacy, Meijo University, 150 Yagotoyama, Tempaku-ku, Nagoya 468-8503, Japan; shuheiy@meijo-u.ac.jp; 2 Center for Medical Genetics, Shinshu University Hospital, 3-1-1 Asahi, Matsumoto, Nagano 390-8621, Japan; ktomoki@shinshu-u.ac.jp

**Keywords:** biglycan, carbohydrate sulfotransferase 14, decorin, chondroitin sulfate, dermatan sulfate, dermatan sulfate epimerase, dermatan 4-*O*-sulfotransferase, Ehlers-Danlos syndrome, glycosaminoglycan, proteoglycan, spondyloepimetaphyseal dysplasia

## Abstract

The indispensable roles of dermatan sulfate-proteoglycans (DS-PGs) have been demonstrated in various biological events including construction of the extracellular matrix and cell signaling through interactions with collagen and transforming growth factor-β, respectively. Defects in the core proteins of DS-PGs such as decorin and biglycan cause congenital stromal dystrophy of the cornea, spondyloepimetaphyseal dysplasia, and Meester-Loeys syndrome. Furthermore, mutations in human genes encoding the glycosyltransferases, epimerases, and sulfotransferases responsible for the biosynthesis of DS chains cause connective tissue disorders including Ehlers-Danlos syndrome and spondyloepimetaphyseal dysplasia with joint laxity characterized by skin hyperextensibility, joint hypermobility, and tissue fragility, and by severe skeletal disorders such as kyphoscoliosis, short trunk, dislocation, and joint laxity. Glycobiological approaches revealed that mutations in DS-biosynthetic enzymes cause reductions in enzymatic activities and in the amount of synthesized DS and also disrupt the formation of collagen bundles. This review focused on the growing number of glycobiological studies on recently reported genetic diseases caused by defects in the biosynthesis of DS and DS-PGs.

## 1. Introduction

**Figure 1:**
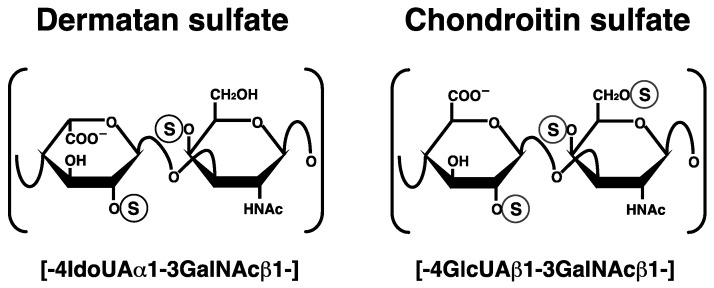
Typical repeating disaccharide units in dermatan sulfate (DS) and chondroitin sulfate (CS), and their potential sulfation sites. The DS backbone consists of l-iduronic acid (IdoUA) and *N*-acetyl-d-galactosamine (GalNAc), whereas CS is a stereoisomer of DS that includes d-glucuronic acid (GlcUA) instead of IdoUA. These sugar moieties may be esterified by sulfate at various positions indicated by the circled ‘S’.

Dermatan sulfate (DS) is a linear polysaccharide that has been classified as a sulfated glycosaminoglycan, which is covalently attached to the core proteins of proteoglycans (PGs) [1,2,3,4]. PGs are widely distributed in extracellular matrices and at cell surfaces [1,2,3,4]. DS-PGs exist abundantly in skin, cartilage, and the aorta. Furthermore, they are ubiquitously expressed in various tissues such as brain, liver, lung, kidney and heart. DS chains consist of alternating disaccharide units comprising L-iduronic acid (IdoUA) and *N*-acetyl-d-galactosamine (GalNAc) residues with 50–200 repeats (Figure 1). DS chains are modified by sulfation at the C-2 and C-4 positions on IdoUA and GalNAc residues, respectively, which is a structural fundament to a wide range of biological events involving DS such as the assembly of extracellular matrices, signal transduction through binding to growth factors, wound healing, and anti-coagulation [2,3]. Chondroitin sulfate (CS) is composed of D-glucuronic acid (GlcUA) and GalNAc. After the synthesis of the chondroitin backbone, the GlcUA residue is epimerized to IdoUA by DS-epimerase (DSE). Thus, the content of IdoUA or DS is varied in each organ or developmental stage. CS-DS hybrid chains are also formed in specific cells and/or tissues.

The small leucine-rich DS-PGs, decorin, biglycan, and fibromodulin, contain leucine-rich regions and small protein cores [1]. The knockout mice of these PGs exhibit skin fragility, and osteoporosis in addition to collagen fibrils with irregular and rough outlines of the Achilles tendon, suggesting that DS-PGs play roles in the formation of skin and bone through the regulation of collagen [5,6,7,8]. Decorin core protein regulates the fibrogenesis of collagen [1]. The knockout mouse of decorin exhibited irregular collagen morphology and similar phenotypes of the human Ehlers-Danlos syndrome (EDS) [5]. In addition, the phenotypes of double-knockout mice of decorin and biglycan directly mimic the rare progeroid variant of the human EDS-like manifestation [9].

EDS is a heterogenous group of heritable connective tissue disorders characterized by skin hyperextensibility, joint hypermobility, and tissue fragility. EDS has been classified into six major types: The classical type (MIM#130000), hypermobility type (MIM#130020), vascular type (MIM#130050), kyphoscoliosis type (MIM#225400), arthrochalasia type (MIM#130060), and dermatospraxis type (MIM#225410) [10,11]. The dominant negative effects of a haploinsufficiency in the genes for the mutant procollagen α-chain or a deficiency in collagen-processing enzymes have been identified as the basis for these types of EDS [10]. Additional forms of EDS have also been reported in association with abnormalities in extracellular matrix proteins and DS [11,12].

This review focuses on DS-defective EDS, which was recently characterized from a glycobiological point of view in terms of disturbances in the biosynthesis of functional DS chains.

## 2. Biosynthesis of DS Chains

### 2.1. Glycosaminoglycan–Protein Linker Region

**Figure 2:**
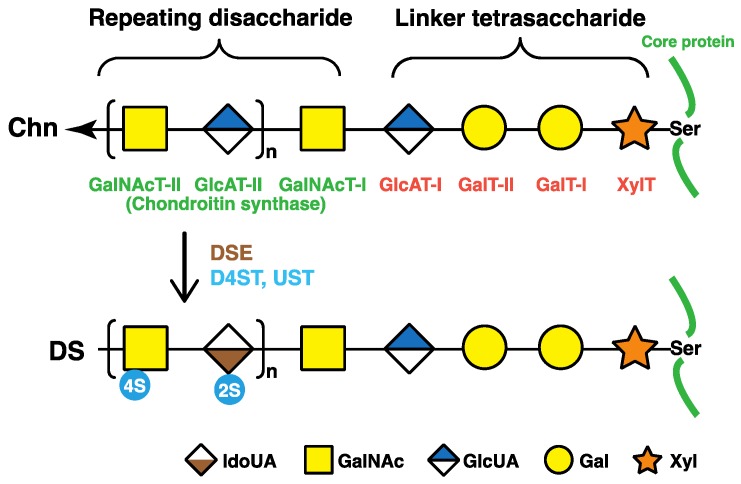
Biosynthetic assembly of DS chains by various glycosyltransferases, epimerases, and sulfotransferases. After specific core proteins are synthesized, the common glycosaminoglycan–protein linker region, GlcUAβ1-3Galβ1-3Galβ1-4Xylβ1-, is built up by XylT, GalT-I, GalT-II, and GlcAT-I on the specific serine (Ser) residue(s) of core proteins. These four groups of enzymes are common to the biosynthesis of glycosaminoglycans including DS, CS, and heparan sulfate. After the formation of the linker region, chondroitin synthases assemble the chondroitin backbone. Thereafter, the epimerization of some GlcUA residues and sulfation of each sugar residue are catalyzed by DSE and D4ST as well as UST, respectively. XylT, β-xylosyltransferase; GalT-I, β1,4-galactosyltransferase-I; GalT-II, β1,3-galactosyltransferase-II; GlcAT-I, β1,3-glucuronosyltransferase-I; GalNAcT-I, β1,4-*N*-acetylgalactosaminyltransferase-I; GlcAT-II, β1,3-glucuronosyltransferase-II; GalNAcT-II, β1,4-*N*-acetylgalactosaminyltransferase-II; DSE, dermatan sulfate epimerase; D4ST, dermatan 4-*O*-sulfotransferase; UST, uronosyl 2-*O*-sulfotransferase; Xyl, d-xylose; Gal, d-galactose; GlcUA, d-glucuronic acid; IdoUA, l-iduronic acid.

The repeating disaccharide regions of DS chains are attached to serine residues in core proteins through the common glycosaminoglycan–protein linker region tetrasaccharide, -*O*-xylose-galactose-galactose-GlcUA- (-*O*-Xyl-Gal-Gal-GlcUA-) (Figure 2) [1]. β-Xylosyltransferases (XylT), which is encoded by *XYLT1* or *XYLT2*, catalyze the transfer of a Xyl residue from uridine diphosphate (UDP)-Xyl to specific serine residues in the core proteins of PGs newly synthesized in the endoplasmic reticulum and cis-Golgi compartments, which initiates the biosynthesis of DS, CS, and heparan sulfate glycosaminoglycan chains (Figure 2) [13,14]. Two Gal residues are added to serine-*O*-Xyl in the core proteins from UDP-Gal by β1,4-galactosyltransferase-I (GalT-I) and β1,3-galactosyltransferase-II (GalT-II), which are encoded by *B4GALT7* and *B3GALT6*, respectively [15,16,17]. β1,3-Glucuronosyltransferase-I (GlcAT-I), which is encoded by *B3GAT3*, then transfers a GlcUA residue from UDP-GlcUA to serine-*O*-Xyl-Gal-Gal (Figure 2) [18].

### 2.2. Repeating Disaccharide Region of DS

The repeating disaccharide region in the chondroitin precursor chain, [-4GlcUAβ1-3GalNAcβ1-]_n_, is constructed by chondroitin synthase family members (Figure 2) [19,20,21,22,23,24]. DSE, which is encoded by *DSE* or *DSE2*, subsequently converts GlcUA into IdoUA by epimerizing the C-5 OH group of GlcUA residues during or after the construction of a chondroitin backbone [25,26]. Dermatan chains are matured by sulfation reactions catalyzed by dermatan 4-*O*-sulfotransferase-1 (D4ST1) and uronosyl 2-*O*-sulfotransferase (UST), which are encoded by *CHST14* and *UST*, respectively. D4ST1 and UST transfer the sulfate group from the sulfate donor 3′-phosphoadenosine 5′-phosphosulfate to the C-4 position of GalNAc and C-2 position of IdoUA residues in dermatan, respectively [27,28,29].

## 3. Human Disorders Affecting the Skeleton and Skin Caused by Disturbances in DS Biosynthetic Enzymes and DS-Proteoglycans

### 3.1. B4GALT7 (GalT-I) Deficiency

Mutations in *B4GALT7*, which encodes GalT-I, cause EDS-progeroid type 1 (Table 1, MIM#130070) [30,31,32,33,34,35]. The characteristics of EDS-progeroid type 1 include an aged appearance, developmental delays, a short stature, craniofacial dysmorphism, generalized osteopenia, defective wound healing, hypermobile joints, hypotonic muscles, and loose yet elastic skin [30,31,32,33]. GalT-I encoded by *B4GALT6* is responsible for the synthesis of the linkage region tetrasaccharide, -Xyl-Gal-Gal-GlcUA-, which is common to both CS/DS and heparan sulfate chains (Figure 2). Fibroblasts from patients with the compound heterozygous mutations of p.Ala186Asp/p.Leu206Pro in GalT-I showed weaker galactosyltransferase activity than that of control subjects, and synthesized de-glycanated decorin and biglycan core proteins in addition to their PG forms with shorter DS chains [33]. Furthermore, a homozygous mutation in *B4GALT7*, p.Arg270Cys, causes the same type of EDS, with patient fibroblasts exhibiting marked reductions in GalT-I activity in vitro and the lack of the DS side chain in 50% of decorin [33]. Fibroblasts with the mutation of p.Arg270Cys show reductions in the sulfation of heparan sulfate chains and the retardation of wound closure in vitro [35]. Thus, various clinical manifestations in EDS-progeroid type 1 may be partially caused by a lack of heparan sulfate as well as DS.

A variant of Larsen syndrome in Reunion Island in France is caused by a homozygous mutation in *B4GALT7* (p.Arg270Cys), and is characterized by multiple dislocations, dwarfism, distinctive facial features, and hyperlaxity [36]. The clinical manifestations of Larsen syndrome are congenital large-joint dislocations and characteristic craniofacial abnormalities including dislocations of the hip, elbow, and knee as well as foot deformities [37]. Therefore, EDS-progeroid type 1 and Larsen syndrome in Reunion Island may share clinical spectra including joint dislocations. However, the reason why these two disorders are caused by the same mutation in *B4GALT7* currently remains unclear. Further analyses are needed in order to elucidate the underlying pathogenic mechanisms.

### 3.2. B3GALT6 (GalT-II) Deficiency

Compound heterozygous mutations (p.Arg6Trp, p.Asp118Alafs*160, p.Met139Ala141del, p.Arg197Alafs*81, or p.Ser309Thr) in *B3GALT6* encoding GalT-II cause EDS-progeroid type 2 (Table 1, MIM#615349) [38]. The recombinant GalT-II mutant enzyme (p.Ser309Thr) exhibits significantly weaker GalT-II activity than that of the wild-type enzyme [38]. Furthermore, compound heterozygous or homozygous mutations (p.Met1?, p.Ser65Gly, p.Pro67Leu, p.Asp156Asn, p.Arg232Cys, and p.Cys300Ser) in *B3GALT6* cause an autosomal-recessive disorder, spondyloepimetaphyseal dysplasia with joint laxity type 1, which is characterized by kyphoscoliosis, clubfeet, hip dislocation, elbow contracture, platyspondyly, and craniofacial dysmorphisms including a small mandible with a cleft palate, prominent eyes, and a long upper lip (Table 1, MIM#271640) [38,39,40]. The skeletal and connective manifestations of EDS-progeroid type 2 and spondyloepimetaphyseal dysplasia with joint laxity type 1 largely overlap, whereas there are no common mutations in *B3GALT6* in these patients [38]. These findings suggest that the levels (amount) or length of glycosaminoglycan chains in patients with EDS-progeroid type 2 or spondyloepimetaphyseal dysplasia with joint laxity type 1 may differ, which gives rise to the number of clinical manifestations in these patients. The recombinant enzymes, p.Ser65Gly-, p.Pro67Leu-, p.Asp156Asn-, p.Arg232Cys-, and p.Cys300Ser-GalT-II exhibit significantly weaker galactosyltransferase activities than that of wild-type GalT-II [38]. Although wild-type GalT-II is expressed in the Golgi, mutant enzymes (p.Met1?, p.Ser65Gly, p.Pro67Leu, and p.Arg232Cys) are located in the cytoplasm and nucleus [38], indicating that the intracellular mislocalization of GalT-II may be partially or fully defective in glycosaminoglycan biosynthesis. Cultured lymphoblastoid cells from patients showed the reduced biosynthesis of heparan sulfate [38].

Malfait et al. identified homozygous missense mutations (p.Asp207His and p.Gly217Ser) and compound heterozygous mutations (p.Ala108Glyfs*163 and p.Asp207His) in *B3GALT6* in patients exhibiting various symptoms similar to those of EDS and spondyloepimetaphyseal dysplasia with joint hyperlaxity [39]. Cultured fibroblasts from the affected individuals synthesized markedly less DS and heparan sulfate. Beighton et al. reported that six patients with spondyloepimetaphyseal dysplasia with joint laxity from South African families had a homozygous missense mutation (p.Thr79Ala) and compound heterozygous mutations (p.Arg6Trp, p.Pro67Leu, or p.Thr79Ala) in *B3GALT6* [40]. Moreover, Alazami et al. detected a homozygous missense mutations (p.Phe186Lue and p.Arg179_Arg180dup) in patients with profound joint laxity, severe kyphoscoliosis, spondyloepimetaphyseal dysplasia, arthrogryposis, and joint dislocation [41].

These findings indicate that partial defects in DS, CS, and heparan sulfate due to mutations in *B3GALT6* affect the development of not only the skeleton, but also the skin with different or a wide range of symptoms in each patient.

### 3.3. DSE Deficiency

A homozygous missense mutation in DSE (p.Ser268Leu) causes EDS musculocontractural type 2 (Table 1, MIM#615539) [42]. Clinical features including hypermobility of the finger, elbow, and knee joints, characteristic facial features, contracture of the thumbs and feet, and myopathy have been observed in a patient [42]. A marked reduction in the epimerase activity of the recombinant mutant DSE (p.Ser268Leu) as well as in the cell lysate from the patient has been demonstrated. Furthermore, a decrease in the biosynthesis of DS is accompanied by an increase in that of CS in the fibroblasts of patient [42]. Although the complete loss of DS from the fibroblasts of patients with *CHST14* mutations has been reported (see 3.4 *CHST14* deficiency) [46], a small amount of DS has been detected in fibroblasts from the DSE-deficient patient [42]. This finding suggests that the mutant enzyme, p.Ser268Leu-DSE, slightly residual enzymatic activity, or DSE2, which is a homologous protein to DSE in humans, partially synthesizes DS in the fibroblasts of patient.

Syx et al. reported another family with a homozygous DSE missense variant (p.Arg267Gly) [43]. The clinical manifestations of the two affected adult sisters showed craniofacial dysmorphic features, congenital clubfeet, long and slender fingers with contracture, muscle weakness, smooth, hyperextensible, and translucent skin, and the formation of large hematomas. No obvious alteration in the architecture of collagen fibrils was detected in DSE-deficient patients [43]. In contrast, CHST14-deficient patients show collagen bundles with collagen fibrils of various diameters, the intermittent presence of small flower-like fibrils, and irregular spaces filled with granulofilamentous deposits [43]. Thus, DSE-deficient patients may have a milder form of the EDS musculocontractural type than CHST14-deficient patients. However, difficulties are associated with describing the characterization due to the limited number of patients with mutations in DSE. Thus, the further characterization of DSE-deficient patients is needed in order to obtain a better understanding of this disorder.

The deficiencies associated with DSE affect the biosynthesis of DS, which implies that DSE and DS-PGs are both essential to the development of skin and bone as well as the maintenance of extracellular matrices in humans. *Dse*^−/−^ mutant mice had fewer IdoUA residues in the skin and affected collagen fibrils [67]. Moreover, DSE appears to be more efficient at forming IdoUA blocks, which are characteristic of DS, than CS-DS hybrid structures, whereas DSE2 is more efficient at forming a CS-DS hybrid structure than IdoUA blocks [26]. These findings indicate that each function of DSE and DSE2 differs, and also that DSE2 may not be able to fully compensate for the loss-of-function observed in DSE.

### 3.4. CHST14 (D4ST1) Deficiency

**Figure 3:**
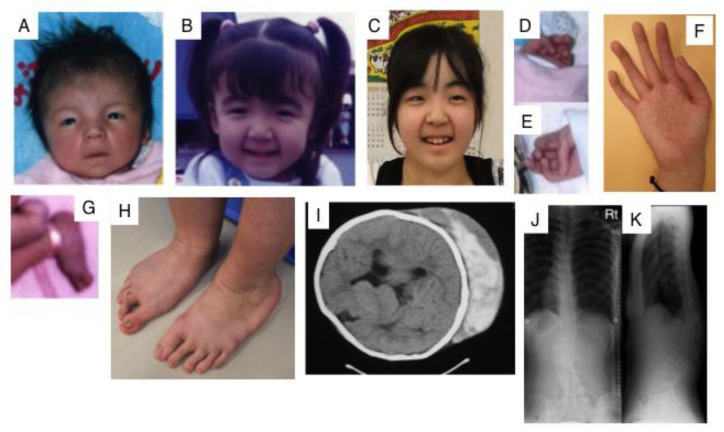
Clinical photographs of a patient with EDS caused by a carbohydrate sulfotransferase 14/dermatan 4-*O*-sulfotransferase-1 (CHST14/D4ST1) deficiency. Craniofacial features at the age of 23 days (**A**); three years (**B**); and 16 years (**C**) indicate hypertelorism; short and downslanting palpebral fissures, blue sclerae, a short nose with a hypoplastic columella, long philtrum, thin upper lip vermilion, small mouth, and micro-retrognathia at birth to early childhood. A slender and asymmetrical facial shape with a protruding jaw from adolescence is also observed. Congenital contracture of the fingers include adducted thumbs at 23 days (**D**,**E**); slender and cylindrical fingers and wrinkling palmar creases at 16 years (**F**); talipes equinovarus at birth (**G**); progressive foot deformities with talipes planus and valgus at 16 years (**H**); large subcutaneous hemtomas in a patient at the age of six years (**I**) and in another patient at the age of 16 years (**J**); kyphoscoliosis in a patient at the age of 16 years (**J**, **K**) (Figure (**I**) was reproduced from [44]; Figures (**A**,**B**,**D**,**E**,**G**,**J**,**K)** were reproduced from [45], with permission from Wiley-Liss, Inc., Hoboken, NJ, USA).

Kosho et al. reported six unrelated Japanese patients with very similar features. They showed multiple congenital malformations, including craniofacial features such as a large fontanelle, hypertelorism, short and downslanting palpebral fissures, blue sclerae, a short nose with a hypoplastic columella, low-set and rotated ears, a high palate, long philtrum, thin upper lip vermilion, small mouth, and micro-retrognathia; multiple congenital contractures including adduction-flexion contracture and talipes equinovarus as well as other visceral or ophthalmological malformations. They also showed progressive multisystem fragility-related complications, including skin hyperextensibility, bruisability, and fragility with atrophic scars; recurrent dislocations; progressive talipes or spinal deformities; pneumothorax or pneumohemothorax; large subcutaneous hematomas; and diverticular perforation (Figure 3). These features are partially similar to those of EDS-kyphoscoliosis type VIA caused by a deficiency in lysyl hydroxylase (Table 1, MIM#601776) [44,45]. Homozygosity mapping of two independent consanguineous families revealed compound heterozygous mutations (p.Lys69*, p.Pro281Leu, p.Cys289Ser, or p.Tyr293Cys) or a homozygous mutation (p.Pro281Leu) in *CHST14*, which encodes D4ST1 [46]. Not only recombinant mutant D4ST1, but also fibroblasts from patients showed a marked reduction in D4ST activity [46]. CS chains, but not non-sulfated DS, have been produced on the decorin of fibroblasts from patients [46]. 4-*O*-Sulfation in CS and DS chains functions as an inhibitor of DSE [68]. Therefore, the defect in D4ST1 allows for a back-epimerization reaction converting IdoUA back to GlcUA to form chondroitin by DSE, followed by the 4-*O*-sulfation of GalNAc residues in chondroitin by C4ST. An aberrant reversed shift from the synthesis of DS to CS may affect the formation or maintenance of adequate collagen bundles in patients [46].

According to Dünder et al., mutations in *CHST14* cause an autosomal recessive disorder, adducted thumb-clubfoot syndrome (Table 1, MIM#601776) [47]. Three homozygous mutations, a 1-bp deletion (p.Val49*), two missense mutations (p.Arg213Pro and p.Tyr293Cys), and a compound heterozygous mutation (p.Arg135Gly and p.Leu137Gln), were found in *CHST14* in the three original adducted thumb-clubfoot syndrome families from Austria and Turkey as well as a consanguineous family from Japan [47]. The adducted thumb-clubfoot syndrome is characterized by adducted thumbs, clubfeet, craniofacial dysmorphism, arachnodactyly cryptorchidism, atrial septal defects, kidney defects, cranial ventricular enlargement, psychomotor retardation, thin and translucent skin, joint instability, and osteopenia from birth to early childhood [48,49]. Five out of eleven patients with the adducted thumb-clubfoot syndrome died in early infancy or childhood, suggesting that adducted thumb-clubfoot syndrome patients may have more severe manifestations than patients with the EDS-Kosho type. Malfait et al. previously reported that a homozygous deletion (p.Val49*) and homozygous 20-bp duplication (p.Glu334Glyfs*107) in *CHST14* from two Turkish siblings and an Indian patient, respectively, caused EDS-musculocontractural type 1 [50].

These disorders are currently considered to be a single clinical entity, with the proposed names ‘D4ST1-deficient EDS’ [51], ‘EDS caused by a CHST14/D4ST1 deficiency’ [12], or ‘EDS, musculocontractural type 1’ [50]. Forty patients from 27 families have been reported to date, including other mutations in *CHST14* (p.Arg29Glyfs*113, p.Gln133Argfs:14, p.Cys152Leufs*10, p.Phe209Ser, p.Arg218Ser, p.Gly228Leufs*13, p.Glu262Lys, p.Arg274Pro, p.Met280Leu, and pTrp327Cysfs*29) [11,12,41,43,44,45,46,47,48,49,50,51,52,53,54,55,56,57,58]. *CHST14* mutations cause a defect in DS side chains on the core protein, decorin, and affect the formation of collagen fibrils. Furthermore, DS side chains on other PGs such as biglycan, versican, epiphycan, endocan, and thrombomodulin may be affected by these mutations, which contribute to the wide range of the clinical manifestations of D4ST1-deficient EDS. Further analyses of the underlying molecular mechanisms may provide a clearer understanding of severe connective tissue disorders, and lead to the development of therapeutic approaches and agents.

*Chst14^−/−^* mice showed smaller body weights, kinked tails, more fragile skin, and lower fertility than wild-type mice [69]. Furthermore, the impaired proliferation of neural stem cells, reduced neurogenesis, and altered subpopulations of radial glial cells were observed in *Chst14^−/−^* mice [70]. These phenotypes are partially consistent with those of patients with D4ST1-deficient EDS. However, biochemical analyses such as the quantification of DS, characterization of DS-PGs, measurement of sulfotransferase activities, and pathological analyses of connective tissues including the skeleton and skin using *Chst14^−/−^* mice have yet be performed. Thus, pharmacotherapeutics for D4ST1-deficient EDS may be accelerated by further analyses using *Chst14^−/−^* mice.

### 3.5. UST Deficiency

A de novo 0.63 Mb deletion on chromosome 6q25.1 causes multiple congenital anomalies such as developmental delay, mild dysmorphic facial features, abnormally elastic and redundant skin, hyperextensible small joints, ventricular septal defect, and underdeveloped cerebellar vermis [59]. This region contains eight genes including *UST*, *TAB2*, and *LATS1*. TAB2 and LATS1 demonstrated to be essential for cardiac development and normal growth, respectively [71,72]. Thus, the patient with ventricular septal defect and developmental delay may be caused by defects in *TAB2* and *LATS1*, respectively. The characteristic joint and skin abnormalities were found in the patient, which are similar to the spectrum of those of the EDS. UST catalyzes the transfer a sulfate group from 3’-phosphoadenosine 5′-phosphosulfate to the C-2 position of IdoUA residues in DS chains [29]. Thus, the loss of *UST* gene may cause EDS-like symptoms.

### 3.6. DCN Deficiecny

Knockout mice of decorin exhibit the EDS-like phenotype [5]. Unexpectedly, mutations found to date in *DCN*, which encodes the decorin core protein, do not cause EDS. All mutations of *DCN* in human cause the truncated form of decorin core protein, resulting in the partial defects in functions of decorin. In fact, a congenital stromal dystrophy of the cornea (MIM#610048) is caused by heterozygous mutations (p.Pro314fs*14, p.Gly316Aspfs*12, p.Lys321Argfs*7, or p.Ser323fs*5,) in *DCN* [60,61,62,63]. Decorin, a prototype small leucine-rich proteoglycan, carries a DS side chain and plays roles in a number of cellular processes including collagen fibrillogenesis, wound repair, angiostasis, tumor growth, and autophagy [1,2,3,73]. This functional diversity may depend on interactions with various extracellular matrix components and growth factors through not only the core protein, but also the DS side chain of decorin. Congenital stromal corneal dystrophy is an autosomal dominant eye disease characterized by corneal clouding with fine opacities observed as small flakes and spots [60,61,64]. Furthermore, affected individuals show refractive errors, amblyopia, strabismus, nystagmus, and esotropia. Although the corneal epithelium and thin basement membrane are normal, not only normal, but also abnormal collagen fibrils have been detected in the stroma lamellae of patients [60,61,62,63,64,73]. The C-terminal truncation associated with mutations in the decorin of patients may affect the interaction with collagen in stroma, which is supported by structural informatics on decorin binding to collagen fibrils [63]. A mouse model of congenital stromal corneal dystrophy has been developed with a frameshift mutation in *DCN*, resulting in a C-terminal truncation lacking 33 amino acids [74]. Mutant mice show corneal opacities, abnormal fibril organization, and greater interfibrillar spaces and fibril diameters. Thus, truncated decorin may affect matrix assembly in the corneal stroma in a dominant-negative manner. Mellgren et al. have reported that that the knock-in mice with the truncated form of DCN exhibited a very low level in cornea, due to accumulation in the endoplasmic reticulum, whereas wild-type DCN was localized in Golgi [75]. Thus, it remains to be elucidated these differences of the phenotypes in both knock-in mice, and why only the corneal stroma is influenced by truncated decorin. The molecular composition of the corneal stroma is mainly type I collagen, which plays an important role in the maintenance of corneal transparency [76]. Therefore, the C-terminal domain of decorin may play roles in organ specific manner or function during the fibrillar organization of cornea.

*Dcn^−/−^* mice show skin fragility and an abnormal collagen morphology [5], which are similar to the symptoms of EDS. Thus, although defects in *DCN* may cause EDS, an EDS patient with a mutation in *DCN* has not been reported to date. Electron microscopic analyses revealed an abnormal morphology and organization of collagen fibrils in the periodontal ligament [77]. The number of periodontal ligament fibroblasts in *Dcn^−/−^* mice was reported to be higher than that in wild-type mice [77], suggesting that decorin regulates cell proliferation. *Dcn*/*Bgn* double knockout mice show severe skin fragility and marked osteopenia, which directly mimics the EDS-progeroid type [9], indicating the potential of this mouse to become a model for the EDS-progeroid type caused by mutations in *B4GALT7* and/or *B3GALT6*.

### 3.7. BGN Deficiency

Two missense mutations (p.Lys147Glu and p.Gly259Val) in *BGN*, which encodes biglycan, cause the X-linked form of spondyloepimetaphyseal dysplasia in Korean, Indian, and Italian families, and are characterized by anomalies of the spine as well as the epiphyses and metaphyses of the long bones, resulting in a short stature and osteoarthritic changes in joints [65] (Table 1, MIM#300106). A member of the small leucine-rich PGs, biglycan, is expressed in bones, and is essential to the assembly of the extracellular matrix and for cell signaling through interactions with type I collagen, transforming growth factor-β (TGFβ), bone morphogenetic protein 4, and Wnt [78,79,80,81]. Mutant proteins do not interact with TGFβ, as shown by an analysis of a molecular dynamics simulation [65]. In addition, the biglycan of fibroblasts from patients is degraded more rapidly than that from unaffected individuals [65]. These findings indicate that the identified variants, Lys147 and Gly259, are located in the leucine-rich region and their substitutions, p.Lys147Glu and p.Gly259Val, may affect decreases in biglycan stability and binding ability with TGFβ.

Meester et al. recently reported that thoracic aortic aneurysms and dissections are caused by mutations (p.Trp2* and p.Gln303Pro) or 21-kb and 28-kb deletions in *BGN* [66] (Table 1, MIM#300989). The clinical manifestation is characterized by the early-onset of aortic aneurysm and dissection, which involves the aortic root or more distal ascending aorta in all patients [66]. In addition, connective tissue features include joint hypermobility and contractures, deformities of skin striae and pectus, craniofacial dysmorphisms including dolichocephaly, hypertelorism, down-slanting eyes, a high-arched plate, proptosis, malar hypoplasia, and frontal bossing as well as the features of Loeys-Dietz syndrome (MIM#609192) such as a bifid uvula and cervical spine instability. Biglycan is not expressed in the aortic walls in affected individuals (p.Gly80Ser and 21-kb deletion). The disorder has been designated as Meester-Loeys syndrome (MIM#300989). The collagen content and elastin fibers in the aortic wall are lower in patients than in healthy controls. Furthermore, increased TGFβ signaling is observed in patients [66], which is similar to affected individuals with Loeys-Dietz syndrome caused by mutations in TGFβ signaling-related proteins such as TGFβ, the TGFβ receptor, and SMAD [82]. Biglycan has also been identified as a regulator of TGFβ signaling [79]. Thus, the clinical phenotypes of individuals with *BGN* mutations are similar to those with mutations in TGFβ-related genes.

*BGN*-deficient mice show a reduced growth rate and decreased bone mass [6]. A BGN deficiency promotes myofibroblast differentiation and proliferation, and this appears to be due to increased responses to TGFβ and SMAD2 signaling [83]. In addition, the aortas of *BGN*-deficient mice show structural abnormalities in collagen fibrils and reduced tensile strength [84], which indicate that biglycan, similar to decorin, is essential to the structural and functional integrity of the aortic wall through the regulation of collagen. These knockout mice may become models for spondyloepimetaphyseal dysplasia and Meester-Loeys syndrome in human *BGN* deficiencies, and may be helpful for developing therapeutic agents for these disorders.

## 4. Conclusions

Recent advances in genetic and glycobiological studies on connective tissue disorders have clarified the biological significance of DS side chains and the core proteins of DS-PGs. Thus, defects in the biosynthesis of DS and DS-PGs may affect the assembly of matrix proteins such as collagen and cell signaling through TGFβ during skeletal and skin formations; however, the underlying pathogenic mechanisms remain unclear. Furthermore, the clinical symptoms of DS-defective genetic disorders are not always similar to the different mutations in DS-biosynthetic enzymes. This number of phenotypes may be partially due to distinct residual functions including the enzymatic activities, cellular mislocalization, or partial compensation by other homologue(s) of each enzyme. A clearer understanding of molecular pathogeneses involving DS chains is essential for facilitating the development of therapeutics for these diseases.

## Figures and Tables

**Table 1:** Human genetic disorders caused by defects in DS chains and core proteins of DS-PGs. B4GALT7, beta1,4-galactosyltransferase 7; B3GALT6, beta1,3-galactosyltransferase 6; DSE, dermatan sulfate epimerase; CHST14, carbohydrate sulfotransferase 14; UST, uronyl 2-sulfotransferase.

Enzymes and DS-PG Core Proteins	Coding Genes	MIM Number	Human Genetic Disorders	Clinical Features	Refs.
β4Galactosyltransferase-I (GalT-I)	*B4GALT7*	130070 604327	Ehlers-Danlos syndrome progeroid type 1	Developmental delays, aged appearance, a short stature, craniofacial dysmorphism, and generalized osteopenia.	[30,31,32,33,34,35,36]
			Larsen of Reunion Island syndrome	Multiple dislocations, hyperlaxity, dwarfism, and distinctive facial features.	
β3Galactosyltransferase-II (GalT-II)	*B3GALT6*	615349 615291	Ehlers-Danlos syndrome progeroid type 2	Sparse hair, wrinkled skin, defective wound healing with atrophic scars, osteopenia, and radial head dislocation.	[38,39,40,41]
		271640	Spondyloepimetaphyseal dysplasia with joint laxity type 1	Spatulate fingers with short nails, hip dislocation, elbow contracture, clubfeet, and mild craniofacial dysmorphism including prominent eyes, blue sclera, a long upper lip, and small mandible with a cleft palate.	
Dermatan sulfate epimerase	*DSE*	615539 605942	Ehlers-Danlos syndrome musculocontractural type 2	Characteristic facial features, congenital contracture of the thumbs and feet, hypermobility of the finger, elbow, and knee joints, atrophic scarring of the skin, and myopathy.	[42,43]
Dermatan 4-*O*-sulfotransferase	*CHST14*	601776 608429	Ehlers-Danlos syndrome musculocontractural type 1; EDS Kosho type	Craniofacial dysmorphism, multiple congenital contractures including adduction-flexion contracture of the thumbs and clubfeet, malformations of the heart, kidney, intestine, and eye; skin hyperextensibility, bruisability, and fragility with atrophic scars; recurrent joint dislocations, progressive foot or spinal deformities, pneumothorax, large subcutaneous hematomas, and diverticular perforation.	[41,43,44,45,46,47,48,49,50,51,52,53,54,55,56,57,58]
			Adducted thumb-clubfoot syndrome		
Uronosyl 2-*O*-sulfotransferase	*UST*	610752	Multiple congenital anomalies of the heart and central nervous system	Growth failure, congenital heart defect, underdeveloped cerebellar vermis, abnormal cutaneous elasticity, and joint laxity.	[59]
Decorin	*DCN*	610048 125255	Congenital stromal corneal dystrophy	Diffuse bilateral corneal clouding, corneal opacities, strabismus, nystagmus, photophobia, and esotropia.	[60,61,62,63,64]
Biglycan	*BGN*	300106 301870	Spondyloepimetaphyseal dysplasia, X-linked	A short stature and osteoarthritic changes in joints; anomalies of the spine, and epiphyses and metaphyses of the long bones.	[65,66]
		300989	Meester-Loeys syndrome	Aortic aneurysm and dissection, hypertelorism, proptosis, downslanting palpebral fissures, frontal bossing, malar hypoplasia, pectus deformities, joint hypermobility or contracture, skin striae, a bifid uvula, cervical spine instability, ventricular dilation, hip dislocation, platyspondyly, phalangeal dysplasia, and dysplastic epiphyses of the long bones.	
